# Early identification of subjective cognitive functional decline among patients with Parkinson’s disease: a longitudinal pilot study

**DOI:** 10.1038/s41598-022-26280-1

**Published:** 2022-12-23

**Authors:** Sara Rosenblum, Sonya Meyer, Ariella Richardson, Sharon Hassin-Baer

**Affiliations:** 1 Department of Occupational Therapy, Faculty of Social Welfare and Health Sciences, University of Haifa, Haifa, 3498838 Israel; 2 Department of Occupational Therapy, Ariel University, Ariel, 4077603 Israel; 3 Department of Industrial Engineering, Jerusalem College of Technology, Jerusalem, 9372115 Israel; 4 Movement Disorders Institute, Sheba Medical Centre, Ramat-Gan, 5262000 Israel; 5 Department of Neurology, Sheba Medical Centre, Ramat-Gan, 5262000 Israel; 6 Sackler Faculty of Medicine, Tel Aviv University, Tel Aviv, 6997801 Israel

**Keywords:** Neurology, Parkinson's disease

## Abstract

Practical methods for early identification of Parkinson’s disease (PD) mild cognitive impairment (PD-MCI) through changes in real-life daily functioning are scarce. The aim of the study was to examine whether the cognitive functional (CF) feature, comprising of seven self-reported Movement Disorder Society’s (MDS) Unified Parkinson’s Disease Rating Scale (UPDRS) items, predicts PD patients’ cognitive functional status after a year. We conducted a 1-year follow-up of 34 PD patients (50–78 year; 70.6% men) suspected of MCI using the following measures: the MDS-UPDRS, UPDRS-CF feature, Beck Depression Inventory (BDI), Montreal Cognitive Assessment (MoCA), Trail Making Test (TMT), Parkinson’s Disease Cognitive Functional Rating Scale (PD-CFRS), and Daily Living Questionnaire (DLQ). The first and second UPDRS-CF feature scores, and additional measures at the 1-year follow-up significantly correlated. Hierarchical regression revealed that the initial MoCA, TMT, and BDI scores predicted the second UPDRS-CF, and the first UPDRS-CF predicted 31% of the second PD-CFRS score variance. Depression moderated the relationship between the first UPDRS-CF score and the DLQ Part A. These results suggest practical, self-reported, daily functional markers for identifying gradual decline in PD patients. They consider the patients’ heterogeneity, underlying cognitive pathology, and implications on daily functioning, health, and well-being.

## Introduction

Cognitive decline is a primary characteristic of mild cognitive impairment (MCI), which is found in about 25% of newly diagnosed patients with Parkinson’s disease (PD) and it may worsen the disease progresses^1^. Despite evidence about the possible negative implications of cognitive decline on daily function, physical and mental health, and quality of life^2,3^, practical methods for identifying PD-MCI through changes in PD patients’ daily functioning have not been established.

When considering the diagnostic criteria for PD-MCI proposed by the Movement Disorder Society (MDS)^4^, cognitive deficits on formal neuropsychological testing should be present, using a two-level testing scheme is proposed, including an abbreviated global cognitive performance test and a second level testing using a comprehensive neuropsychological battery. Additionally, a “gradual decline in cognitive ability” should be reported as well as “existence of cognitive deficits that do not significantly interfere with functional independence”, based on the patient’s or informant/clinician’s subjective report.

Despite the growing literature on PD-MCI, by 2022 and the understanding that cognitive impairment in PD might have a different relationship pattern to the motor and some non‑motor symptoms, PD-MCI still is not clearly defined—especially the impact of these cognitive impairments on patients’ day-to-day functioning^5–7^. Specifically, matters still arise about the extent of “gradual decline” and which questions to ask the patient to achieve evidence of “cognitive deficits which do not significantly interfere with functional independence.” Further, practical standardized tools to facilitate and enhance the accuracy of patients’ reports related to their cognitive abilities in their daily functioning have not been established.

There is a time delay between the time the patient initially recognizes a change in daily functional abilities (i.e., the start of the “gradual decline”) and the time he or she reports it to the treating neurologist. Generally, people perceive physicians as responsible for *physical* health and not their daily functional abilities as their functional needs tend to be ignored by professionals^8^. However, this delay may be critical. Studies have shown that PD patients' performance abilities in activities of daily living (ADL) mediate the relationship between depression and health-related quality of life^9^. Harrison et al.^10^ found a strong correlation between the Unified Parkinson’s Disease Rating Scale (UPDRS) ADL sub-score^11^ and disease duration among PD patients. This finding suggested that the ADL section might measure disease progression better than the other UPDRS sections.

Formulated on these results, in our first study on 78 PD patients suspected for PD-MCI, we chose seven items from the MDS-UPDRS regarding non-motor and motor aspects of PD to create a UPDRS-cognitive-functional feature (UPDRS-CF). These included 1.1 cognitive impairment, 2.1 speech, 2.4 eating, 2.5 dressing, 2.6 hygiene, 2.7 handwriting, and 2.8 doing hobbies and the UPDRS-CF was calculated as the mean score of these items^12^. We found no correlations between the Montreal Cognitive Assessment MoCA)^13^ scores and both self-reported questionnaires: the cognitive failure questionnaire (CFQ)^14^, the Parkinson’s Disease Cognitive Functional Rating Scale (PD-CFRS)^15^ scores, nor with the UPDRS-CF score. However, significantly positive correlations were found between the UPDRS-CF and both the self- reported CFQ score and the PD-CFRS score. Furthermore, a significant medium correlation was found between the CFQ and the PD-CFRS scores^12^. A problem was shown in this study regarding the compatibility of the MoCA^13^ used as a scale for global cognitive abilities (Level I criteria) and neuropsychological tests based on cutoff scores (Level II criteria). These issues challenge the clinical application of the MDS diagnostic criteria for PD-MCI and its relatedness to real-world daily functioning. It is questionable whether the MoCA or neuropsychological tests considered “objective” measures^12,16^ indeed timely capture people’s mild cognitive decline in their daily functioning as reflected by subjective self-reports. In addition, the MoCA may be susceptible to practice effects, when administering more than once, as performed in follow-up^17^. The tendency to seriously consider the patients self-report was further exhibited in additional analysis of the same 86 PD patients who were suspected for PD-MCI^18^. Significant high correlations were found between the UPDRS-CF feature and the PD-CFRS^15^, Daily Living Questionnaire (DLQ(^19^, and Time Organization and Participation Scale (TOPS)^20^ scores^12^. Both the DLQ and TOPS enable patients to report about their daily cognitive functional confrontations.

Thus, we assumed that the UPDRS-CF feature may be an early sign for PD-MCI. Indeed, when patients were divided into two groups based on their UPDRS-CF feature, one as suspected for MCI (s.MCI; n = 25) and one as not (PD; n = 53), significant between-group differences were found in their standardized cognitive functional self-report questionnaires scores (DLQ and TOPS). Moreover, specific questionnaires items accounted for 35% of the variance in the UPDR-CF feature, which correlates with daily cognitive functional states^18^.

Importantly, these cognitive functional scales are based on the patient’s subjective report. Although Galtier and colleagues^21^ indicated that the clinical value of such subjective cognitive decline (SCD) in PD (PD-SCD) is still unclear, their long-term study results noted PD-SCD as a risk factor for later developing dementia. Previous studies also indicated that self-reported subtle cognitive and functional decline indeed predicts future meaningful cognitive and functional changes and depression levels among PD patients^22,23^. In their systematic review and meta-analysis, Saredakis and colleagues^24^ described the trajectory of cognitive impairment in PD. They found that within 3 years of follow-up, 25% of the PD patients declined from normal cognition to MCI, and 20% of PD-MCI patients to dementia^24^. Thus, while considering the duration, a 1-year follow-up study that included 34 of our PD patients suspected of MCI using the same measures of health (the MDS-UPDRS^11^ and Beck Depression Inventory [BDI]^25^, MoCA, TMT^13,26^, and daily cognitive functional abilities (UPDRS-CF, PD-CFRS, and DLQ.A) was performed.

The aim of this study was to look at changes of both cognitive and functional measures during a 1-year period, focusing on our suggested UPDRS-CF feature.

## Methods and materials

### Participants

The current study included 34 patients diagnosed with probable or possible PD according to the MDS clinical diagnostic criteria^27^ within up to 10 years of the PD duration. They represented a partial group of the 119 participants in a larger study^12^. Inclusion criteria were age 40 to 80 years; resided in Israel for at least 10 years; could speak, write, and read Hebrew; and had normal or corrected vision and hearing ability. Only patients with Hoehn and Yahr Stage 1 or 2^27,28^ were included. Exclusion criteria were any other neurological condition (e.g., brain trauma, tumor, or surgery; stroke or epilepsy), psychiatric disease or significant systemic disease. Patients with MDS-UPDRS cognitive impairment (1.1) and depressed mood (1.3) scoring 3 or more on either were excluded. Patients included had to score 18 or lower on the BDI^25^ and 23 or more on the MoCA^29^, were functionally independent, and lived in private homes or assisted-living facilities. The 34 patients included in the current sample were chosen for follow-up as they were a subgroup who participated in testing a mobile application tested over time as part of the study^30^.

### Ethical considerations

This study was approved by the of Institutional Ethics Committee of Chaim Sheba Medical Center (protocol code 3852-17-SMC and date of approval 30 March 2017). Written informed consent was obtained from each participant. All procedures involving human participants were performed in accordance with the ethical standards of the institutional and/or national research committee as well as the tenets of the 1964 Declaration of Helsinki and its later amendments, or comparable ethical standards.

### Procedure

Participants were invited to the Movement Disorders Institute for two evaluations—the first (Time1) at the start of the study and the second a year later (Time2). A movement disorders neurologist assessed the participants’ PD-related symptoms and signs using the patients’ self-reports, physical examinations, and MDS-UPDRS^11^. The neurologist rated the patients during their “on” state (i.e., when patients have taken their dopaminergic medication).

We administered the MoCA and a battery of neuropsychological tests to all the participants^13^. PD-related clinical and demographic data, medical comorbidities, and PD medication documentation from patient files and calculated each patient’s total daily Levodopa equivalent dose.

### Measures

#### Neuropsychological and cognitive assessments

Based on the results of a previous study^12^, we included the TMT^26^ as a neuropsychological test in the current study. We used the TMT.A to measure attention and the TMT.B to measure EF (working memory). Further, we used the MoCA to test global cognitive abilities^13^.

#### Self-report cognitive function questionnaires

A certified occupational therapist provided and administered the self-report questionnaires, which focused on the participants’ functional cognition. We used Parts I and II of the MDS-UPDRS^11^ to retrieve patient-reported dysfunction associated with cognitive procedures in motor and nonmotor daily experiences. The UPDRS-CF was calculated as the mean score of seven MDS-UPDRS items chosen for this study: (1.1) cognitive impairment, (2.1) speech, (2.4) eating, (2.5) dressing, (2.6) hygiene, (2.7) handwriting, and (2.8) doing hobbies.

#### Parkinson’s disease cognitive functional rating scale (PD-CFRS)

The PD-CFRS is a reliable, valid, 12-item self-report questionnaire exploring a range of functional aspects sensitive to early and mild cognitive decline in PD^15^ relative to activities. Scores can range from 0 to 2, with higher scores indicating greater decline. We implemented a Hebrew version in this study with the original author’s approval. Example items include: “Have you had trouble handling doctor appointments, bills, or personal mail? Arranging your holidays? Meeting with family or friends?”.

#### Daily living questionnaire

The DLQ includes 52 items scored on a 5-point Likert scale to evaluate functional cognition^19^. Respondents rate whether they have mental difficulty for each item as 1 (*no difficulty*), 2 (*some difficulty*), 3 (*much difficulty*), 4 (*unable to do*), or 0 (*not applicable/ not rated*). Part A, Activities and Participation, includes the subscales of activities involving language/comprehension, community/participation, and household and complex tasks. Example items include finding items on crowded shelves or in the closet, shopping (e.g., buying what you need, making decisions, finding items), and household tasks (e.g., organizing laundry). Part B, Cognitive Symptoms of Impairments, includes the subscales of EF and EF monitoring and memory. Example items include understanding new information, remembering things you need to do during the day, and attending to all aspects of a task or situation without missing information. Internal reliability in the current sample was α = 0.95 (DLQ.A) and α = 0.96 (DLQ.B).

### Statistical analysis

Analysis was performed using IBM SPSS Statistics software (version 27; IBM Corp., Armonk, NY). Statistical assumptions for all analyses were tested to ensure accurate interpretation of results. The level of statistical significance was set at *p* = 0.05.

Descriptive statistics were used to describe the sample. We checked the internal reliability of each self-report questionnaire’s domains (Cronbach’s alpha) at the first and second evaluations. We conducted Pearson correlations and due to multiple correlations, Bonferroni correction was applied. For the initial correlation analysis between the first and second evaluation scores of the UPDRS-CF features, MoCA, neuropsychological tests, and the BDI, a *p* ≤ 0.02 was established as significant. For the next correlation between the UPDRS.CF.1 and UPDRS.CF.2 features and the CFRS.2 and DLQ.A.2 scores, *p* ≤ 0.01 was determined as significant.

Differences between the first and second UPDRS subscale scores, MoCA and BDI were analyzed by repeated measures ANCOVA, while holding gender and PD onset age as covariates. Hierarchical regression was conducted to measure the first UPDRS.CF feature’s prediction of the CFRS.2 and DLQ.A.2 scores, while gender and onset age were included at the first step. We used the Hayes Process Model 1 to test whether depression (BDI) moderates the relationship between the UPDRS.CF.1 feature and both CFRS.2 and the DLQA.2 scores and defined gender and onset age as covariates.

## Results

### Participants’ demographic characteristics and medical status

Thirty-four participants diagnosed with PD with suspected PD-MCI were included in the follow-up study: 24 (70.6%) men and 10 (29.4%) women, aged 50 to 78 yr (*M* = 63.59 yr, *SD* = 7.66). None of the patients were taking medication for dementia (neither choline esterase inhibitors nor memantine), and none were taking antipsychotics. Further demographic and medical status characteristics are described in Table 1. The repeated measures ANCOVA, while gender and age onset were held as covariates, indicated no significant differences between the first and second UPDRS total score (F(1,31) = 3.43, *p* = 0.074), UPDRS * gender (F(1,31) = 0.63, *p* = 0.43, UPDRS * age onset (F(1,31) = 3.64, *p* = 0.07). No significant differences were found between the first and second MoCA scores, (*F*(1,29) = 0.010, *p* = 0.92). Significant differences were found for MoCA*gender (F(1,29) = 4.88, *p* = 0.035) showing a significant effect for MoCA*gender but not for MoCA*age onset (F(1,29) = 0.002, *p* = 0.97). When comparing the first and second BDI score, no significant differences were found (F(1,29) = 0.15, *p* = 0.70) or BDI*gender (F(1,29) = 0.34, *p* = 0.56) and for BDI*age onset (F(1,29) = 0.01, *p* = 0.93).

**Table 1:** Demographic and medical status characteristics of parkinson’s disease patients suspected for PD-MCI at the start and 1-year follow-up.

Variable	Statistic (*N* = 34)	
	Frequency (%)	
Gender (male)	24 (70.6%)	
Country of birth (Israel)	24 (70.6%)	
Patients treated with Levodopa	16 (48.0%)	
	*M* (*SD*)	
Age (yr) *M* (SD)	63.59 (7.66) Range: 50–78	
PD onset age	59.34 (8.52) Range: 42–75	
Education (yr)* *M* (SD)	14.74 (3.87) Range: 0–22	
PD duration since diagnosis (yr) *M* (SD)	4.24 (3.33) Range: 1–12	
LED (Mg)* *M* (SD)	483.49 (427.95)	
Hoehn & Yahr stage “on”*	Range: 1.0–2.5 Median: 2	
	First evaluation	Second evaluation
	*M* (*SD*)	
MoCA score	24.91 (2.00) Range: 22–29	23.97 (2.94) Range: 18–29
BDI score	7.71 (5.32) Range: 1–22	9.59 (7.05) Range: 1–25
MDS-UPDRS P-I	7.09 (5.21)	8.06 (6.35)
MDS-UPDRS P-II	9.29 (5.42)	10.03 (6.24)
MDS-UPDRS P-III	22.35 (9.34)	24.50 (10.86)
MDS-UPDRS P-IV	2.24 (3.69)	2.71 (3.34)
MDS-UPDRS total	40.97 (16.73) Range: 14–73	45.28 (20.96) Range: 19–83

LED, total daily Levodopa equivalent dose; Mg, milligrams; MoCA, Montreal Cognitive Assessment; BDI, Beck Depression Inventory^25^ MDS-UPDRS P-I, nonmotor experiences of daily living; MDS-UPDRS P-II, motor experiences of daily living; MDS-UPDRS P-III, motor examination; MDS-UPDRS P-IV, motor complications ^4^.

### Correlations

Significant correlation was found between the first and second MoCA scores (*r* = 0.431, *p* = 0.014). A significant high correlation (*r* = 0.782, *p* < 0.001) was found between the first and second UPDRS-CF results. Table 2 presents significant correlations between the first UPDRS-CF.1 feature with both TMT.2 components. The TMT.B.2 significantly correlated with the UPDRS-CF.1 feature.

**Table 2:** Correlations between the first and second UPDRS-CF and second MoCA and neuropsychological test scores (TMT).

Score	MoCA.2 score	TMT.A.2	TMT.B.2
UPDRS-CF.1 (initial)	− .359	.401*	.441**
UPDRS-CF.2 (1 year)	− .136	.236	.363

*Correlation is significant at *p* ≤ .02 after Bonferroni correction; **p < .01.

Significant correlations were found between the first and second UPDRS-CF features and participants’ functional cognitive questionnaires scores (CFRS, DLQA, DLQB) at the 1-year follow-up (Table 3).

**Table 3:** Correlations between the first and second MoCA scores, UPDRS-CF features and participants’ second BDI and functional cognitive questionnaires scores at 1-year follow-up.

Score	BDI.2	PD-CFRS.2	DLQ.A.2	DLQ.B.2
MoCA.1	− .187	− .157	− .099	− .118
MoCA.2	− .142	− .271	− .236	− .233
UPDRS-CF.1 (initial)	.407	.662***	.733***	.591***
UPDRS-CF.2 (1 year)	.542**	.602***	.626***	.521**

BDI, Beck Depression Inventory^25^; PD-CFRS, Parkinson’s Disease Cognitive Functional Rating Scale^15^; DLQ, Daily Living Questionnaire: A, activities and participation, B, cognitive domains^19^.

**Correlation is significant at *p* ≤ .01 level after Bonferroni correction; *** *p* ≤ .001.

### Predictions

The hierarchical regression to predict the PD-CFRS.2 included gender and age onset at the first step, the MoCA.1 score at the second step, the TMT.B.1 score at the third step, the BDI at the fourth step, and lastly the UPDRS.CF.1 score. The TMT-A score was not included as we found multicollinearity between TMT-A and B while, as presented in Table 2, TMT-B had a higher level of correlation with the PD-CFRS.2 and DLQ.A.2 scores.

Results indicated that only the UPDRS-CF.1 predicted 31% of the variance of the PD-CFRS.2 score (Table 4).

**Table 4:** Predicting the CFRS.2 score by gender and PD onset, the First MoCA.1, TMT.B.1, and BDI.1 scores and UPDRS-CF.1 Feature.

Variable	Model 1			Model 2			Model 3			Model 4			Model 5		
	*B*	*SE B*	β	*B*	*SE B*	β	*B*	*SE B*	β	*B*	*SE B*	β	B	*SE B*	β
Gender	2.02	3.48	.10	1.92	3.58	.10	1.15	3.64	.06	3.04	3.62	.15	1.99	2.83	.10
Age onset	.33	.20	.29	.31	.22	.27	.38	.23	.39	.45	.22	.40	.37	.17	.33
MoCA.1				− .15	.91	− .03	.18	.95	.04	.59	.93	.13	− .41	.76	− .09
TMT.B.1							.65	.57	.22	.71	.55	.24	− .44	.51	− .15
BDI.1										.60	.31	.35	− .07	.29	− .04
UPDRS.CF.1													12.26	2.92	.75***
*R*^2^	.10			.10			.14			.25			.56		
*F* change in *R*^2^	1.53			.03			1.29			3.75			17.62***		

***p < .001.

The hierarchical regression to predict the DLQ.A.2 included the gender and PD onset at the first step, MoCA.1 score at the second step, the TMT.B.1 score at the third step, the BDI at the fourth step, and the UPDRS.CF.1 score last.

Results indicated that the TMT.B predicted 24% of the DLQ.A.2 variance, The BDI.1 contributed an additional 14%, and UPDRS-CF.1 added another 20% to the prediction. All in all, those measures predicted 59% of the DLQ.A (Table 5).

**Table 5:** Prediction of Follow-Up DLQ.A.2 Score by gender, age onset, the MoCA.1, TMT.A.1, TMT.B.1, BDI.1, and UPDRS-CF.1 Feature.

Variable	Model 1			Model 2			Model 3			Model 4			Model 5		
	*B*	*SE B*	Β	*B*	*SE B*	β	*B*	*SE B*	β	*B*	*SE B*	β	*B*	*SE B*	Β
Gender	.08	.19	.08	.06	.19	.06	-.03	.17	− .03	.08	.17	.08	.03	.14	.03
Age onset	.00	.01	.01	− .00	.01	− .03	.01	.01	.12	.01	.01	.19	.01	.01	.13
MoCA.1				− .02	.05	− .09	.02	.04	.08	.04	.04	.18	.00	.04	.00
TMT.B.1							.08	.03	.53**	.08	.02	.56**	.04	.02	.24
BDI.1										.03	.01	.40*	.01	.01	.08
UPDRS.CF.1													.51	.14	.61**
*R*^2^	.01			.01			.25			.39			.59		
*F* change in *R*^2^	.09			.22			8.70**			5.89*			12.42**		

*p < .05, **p < .01, ***p < .001.

**Figure 1:**
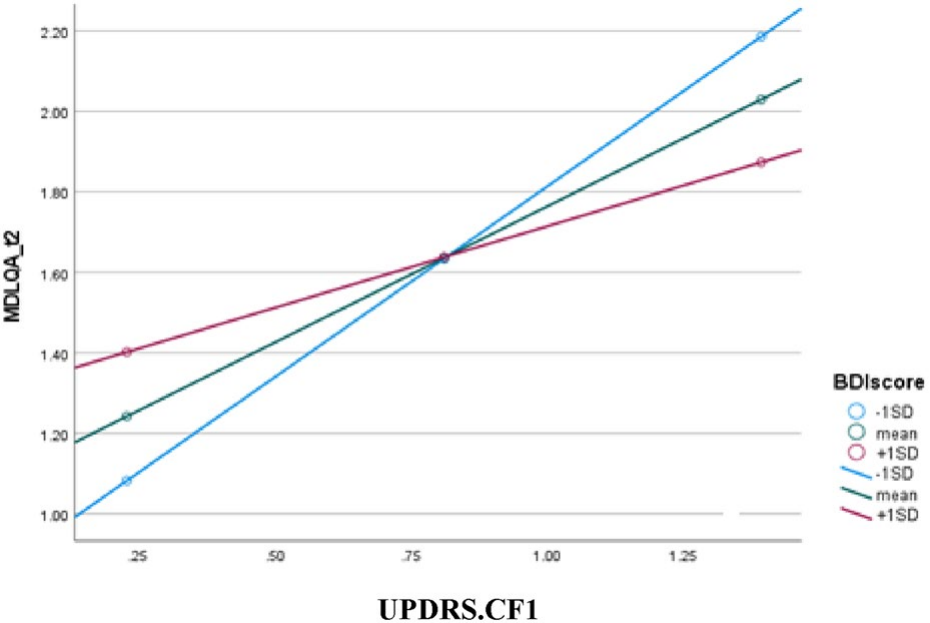
Moderation effect model—relationships between first UPDRS.CF.1, BDI and DLQA.2 while age and years with PD were entered as covariates.

Results of the Hayes Process Model 1 revealed that when taking gender and age onset as covariates, depression (BDI) moderates the relationship between UPDRS-CF.1 and DLQ.A.2 (β = − 0.051, *SE* = 0.0187, *t* = 2.725, *p* = 0.011), such that at lower depression levels, the effect of the UPDRS-CF.1 on the DLQ.A.2 is stronger (Fig. 1).

## Discussion

This study’s results contribute to the ongoing discussion of the challenge of capturing markers for subtle cognitive functional decline among PD patients that may worsen their future mental and physical health^31^.

Over a year, although there was a decrease in the MoCA score, this decrease was significant only when taking gender into consideration in the comparison, indicating lower global cognitive abilities only among the women. Further studies are required to profoundly examine this difference due to the very limited number of women in the current sample. Despite the higher BDI^32^ mean score, showing higher depression levels, these results were not significant. Such results hint towards the dynamic process that occurs with time. They raise questions about possible relationships among the participants’ inferior cognitive abilities, functional status, and mental health^2,3^.

Interestingly, participants’ UPDRS-CF features, a practical and efficient measure, continued to constitute a marker for their cognitive functional abilities, given the significant high correlation found between their first and second UPDRS-CF features. The fact that no significant correlations were found between the first and second MoCA scores and the self-reported functional cognition questionnaires but found with the first and second UPDRS-CF feature scores emphasize the uniqueness of this simple practical measure as a possible early marker of PD-MCI. In fact, both the UPDRS-CF feature and the self-report cognitive functional questionnaires reflect the gradual cognitive decline which patients feel in their daily function and able to express when asked questions about real-life daily function nuances. As a reflection of daily cognitive functional abilities, the UPDRS-CF feature not only exhibited significant medium correlations between its first and second features but also between both those features and the self-reported cognitive functional scales scores (PD-CFRS and DLQ). When trying to capture small nuances of changes in daily function, the questions to be asked becomes crucial. The uniqueness of the PD-CFRS and the DLQ.A self-report questionnaires is their focus on the manifestations of integrating cognitive abilities in day-to-day functional activities^19^. It is worth mentioning that the TMT.B, UPDRS-CF, and BDI predicted scores of both the PD-CFRS and DLQ.A.

These results reinforce the importance of the patients’ self-reports about both their cognitive and ADL functional abilities. They further support Harrison et al.’s^10^ findings of the greater sensitivity of the UPDRS-ADL than other UPDRS sections as a PD progression marker. Further evidence of this marker’s importance is the significant correlations between the first UPRS-CF.1 feature and the first and second TMT scores^26^. As a neuropsychological test, the TMT’s relation to functional status among PD patients and older adults has been described previously. For instance, Higginson and colleagues^33^ found that the Parts A and B scores of the TMT contributed to predicting abilities to complete instrumental ADL among PD patients. Mitchell and Miller^34^ found the TMT to be the only measure of executive functioning that accounted for functional status among older adults.

Interestingly, in that context in our study, significant correlations were found between the first UPDRS-CF.1 feature and both TMT.A.2 and TMT.B.2. The significant correlations found between both TMT parts in the second year with the UPDRS-CF feature, reinforce this performance-based measure’s strength in reflecting the participants’ cognitive functional status required in real-life daily tasks performance. Other tools may supplement the benefits of those markers among PD patients. For example, performing the TMT on a Computerized Penmanship Evaluation Tool^35^ may add objective spatial–temporal and pressure performance measures to support PD-MCI diagnoses in future studies^36,37^. The results of this study support the importance of the UPDRS-CF feature as a predictor of MCI, thus highlighting the significance of identifying subjective functional cognitive decline^38^.

The literature hosts a debate over the need for “objective” versus “subjective” measures of the patient’s cognitive abilities. *Objective* means cognitive and neuropsychological tests; *subjective* means the patient’s complaint/report, as the UPDRS-CF, PD-CFRS, and the DLQ provided in this study (e.g.,^2,22^).

Copeland and colleagues’^16^ support the importance of subjective report found in our study. The researchers administrated a neuropsychological battery to serve as an objective battery. Their subsequent interview questions related to attention, memory, language, and visuospatial abilities. They found no correlations between the objective and subjective reports but found consistency between the patients’ and their caregivers’ reports. When focusing on subjective and objective EF measures among PD patients, Korets and colleagues^39^ found a dis-correlation between PD patients’ reports based on the Dysexecutive Questionnaire measuring EF manifestations in daily life (subjective) and the objective Frontal Assessment Battery (FAB). Their findings indicated that not all PD patients who showed EF impairments in the FAB reported them in the Dysexecutive Questionnaire, and not all PD patients who reported EF problems showed impairments on the FAB.

Galtier and colleagues’^21^ follow-up study showed that 33% of the PD patients who reported subjective cognitive decline (PD-SCD) and then developed dementia, compared to 14.3% among those without subjective cognitive complaints and 50% of those diagnosed with PD-MCI. Further, Galtier et al.^21^ found no significant differences between the control and PD-SCD groups in any neuropsychological measure. More recently, Ophey and colleagues^40^ used various standardized, self-report SCD questionnaires to indicate cognitive decline reflected in daily situations and correlated early brain pathology with the SCD reports.

Such results raise the question of whether “objective” measures indeed reflect real-world functional cognition. The neuropsychological tests measure separate cognitive domains, but life is much more demanding—it requires using multiple cognitive domains simultaneously^41^. Based on the results of these studies, it may be assumed that PD patients feel changes in the cognitive functional abilities that require integrating multiple brain functions in the real world—even before neuropsychological tests examining discrete brain functions reflect those changes.

Furthermore, a vast variability occurs among PD patients^42^. The same minimal cognitive deficit in specific or multiple domains may have varied implications on each individual’s functional abilities, depending on the individual’s environmental expectations and coping style^43,44^. For example, a slight decline in attention abilities may influence each individual’s gait control differently according to the tasks they need to perform, their environmental expectations, and their motor and emotional control^45^.

The complex process of understanding each individual’s unique day-to-day functional experience is crucial for implementing interventions based on person-centered goal setting and improving physical and emotional well-being^46,47^. However, in their comprehensive review, Oedekoven and colleagues^48^ concluded that despite evidence for the possible benefits of SCD for early identification of PD-MCI, no consensus exists for SCD research or clinical criteria.

The high significant correlation found between the UPDRS-CF feature and the BDI in the second year is an important finding. The UPDRS-CF feature includes the participants’ cognitive *and* functional status: As the cognitive and functional status decrease (reflected in higher scores), the individual’s emotional status decreases (shown by higher depression levels). This means that early identification of the decreased functional status and supplying the appropriate functional intervention may prevent mental health disease and the worsening of cognitive abilities towards dementia^24^. The results of the model presented at least reinforce the importance of the daily cognitive functional status. Although no significant moderation was found for the BDI to the PD-CFRS questionnaire score, the BDI indeed moderated the relationship between the UPDRS-CF.1 and DLQ.A.2. Specifically, at lower depression levels (BDI), the effect of the UPDRS-CF.1 on the DLQ.A.2 is stronger. As such, identifying and reinforcing daily functional abilities may prevent depression.

Both regression analyses spotlighted the meaning of the relationships between deficient functional cognitive abilities and mental health among PD-MCI patients. In both regressions, the UPDRS-CF.1 added a considerable percentage to the variance prediction of the functional cognitive self-report results.

Relative to predicting the DLQ.A.2, the BDI score also contributed 6%. In addition, a high percentage was predicted by the TMT test reflecting EF, visual-spatial processing, working memory motor speed, and sequencing with cognitive flexibility. shedding light on the possible mechanism influencing the patients’ day-to-day behavior and health status.

Although declines in memory, visuospatial, and attention/executive abilities were common among PD-patients with MCI^49^, there is a lack of knowledge about their early manifestations or other cognitive deficits in the primary phases of cognitive decline among this population. Identifying PD-MCI as reflected in changes by their daily functional abilities on self-reports and performance-based tasks such as the TMT provides an opportunity to understand cognitive decline in PD and its progression to dementia and poor mental health^40,50,51^.

## Limitations

This study’s main limitations relate to sample size, type, and length. Most of the 34 participants were men, and the follow-up study was conducted after 1 year. Further longitudinal studies with larger, more representative samples, also including PD patients with no MCI diagnosis and performed over longer periods are required to remeasure the standardized cognitive functional measures for early identification and prediction of PD-MCI.

## Conclusion

Our primary study results suggest practical, self-reported, daily functional markers for identifying the decline. They enable considering the vast heterogeneity among PD patients, including their underlying cognitive pathology and its implications on their daily function, physical and emotional health, and well-being^16^. Better insight and awareness of cognitive functional decline as an indicator of PD-MCI may have future therapeutic implications for improving this population’s life quality^52^.

## Data Availability

Restrictions apply to the availability of the data that support the findings of this study, which were used under license and so are not publicly available. However, the data are available from the authors upon reasonable request and with permission of the fourth author.
